# Breathless Revelation: Unmasking Acute Myeloid Leukemia Through Acute Respiratory Failure

**DOI:** 10.7759/cureus.49073

**Published:** 2023-11-19

**Authors:** Seth Lisle, Ekrem Yetiskul, Yisroel Y Grabie, Sudeep Acharya, Michel Chalhoub

**Affiliations:** 1 Internal Medicine, Touro College of Osteopathic Medicine, New York, USA; 2 Internal Medicine, Staten Island University Hospital, Staten Island, USA; 3 Internal Medicine/Pulmonary and Critical Care, Donald and Barbara Zucker School of Medicine at Hofstra/Northwell, Staten Island, USA; 4 Pulmonary and Critical Care Medicine, Staten Island University Hospital, Staten Island, USA

**Keywords:** community-aquired pneumonia, interstitial pneumonia, severe sepsis, acute hypoxemic respiratory failure, acute myeloid leukemia (aml)

## Abstract

Establishing a diagnosis of acute myeloid leukemia (AML) in a patient presenting with acute respiratory failure is rare. Here, we present a case of AML initially appearing as hypoxemic respiratory failure linked to presumed community-acquired pneumonia. This case report unravels the intricate diagnostic odyssey of an atypical AML presentation masquerading as an acute respiratory failure, accentuating the multifaceted challenges clinicians encounter in discerning the actual underlying pathology amidst the haze of mimicry. Upon meticulous diagnostic expedition, infection was ruled out as a cause of respiratory failure, and the patient underwent a malignancy workup, ultimately culminating in the diagnosis. This case underscores the importance of broader diagnostic vigilance. Comprehensive assessments, combined with interdisciplinary collaboration, emerged as crucial for accurate diagnosis, emphasizing the need to consider hematologic pathologies despite seemingly unrelated clinical presentations.

## Introduction

Acute myeloid leukemia (AML) is the most common leukemia among the adult population and accounts for about 80% of all cases. It is characterized by clonal expansion of immature "blast cells" in the peripheral blood and bone marrow, resulting in ineffective erythropoiesis and bone marrow failure [1]. Symptomatic leukemic infiltration of the lung is the least common cause of pulmonary infiltrates in patients with acute leukemia [2]. A retrospective study reported leukemic infiltration to account for (3.8%) of pulmonary complications in AML. Infection was the most common cause of pulmonary complications in AML; although most cases represent infectious pneumonia, with bacteria (28.3%) and fungi (26.5%) as the most prevalent pathogens, noninfectious etiologies such as pulmonary embolism (7.5%), pneumothorax (3.8%), and cardiac disease (9.4%) also must be taken into account [3]. AML can be diagnosed incidentally or may present with non-specific constitutional symptoms (e.g., fatigue and pallor, secondary to anemia). This case chronicles the diagnostic journey of a 60-year-old former firefighter involved in 9/11 rescue operations. His initial presentation of shortness of breath, suggestive of community-acquired pneumonia, resulted in hemodynamic instability requiring ICU-level supportive care, eventually leading to the unexpected diagnosis of AML.

## Case presentation

**Figure 1:**
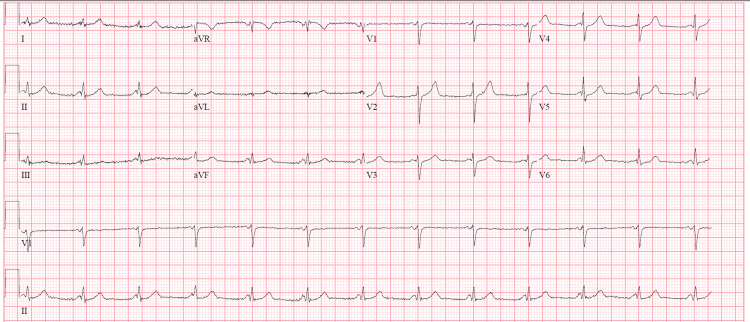
EKG on admission, demonstrating sinus rhythm with short PR, low-voltage QRS, possible inferior infarct, and age undetermined.

**Figure 2:**
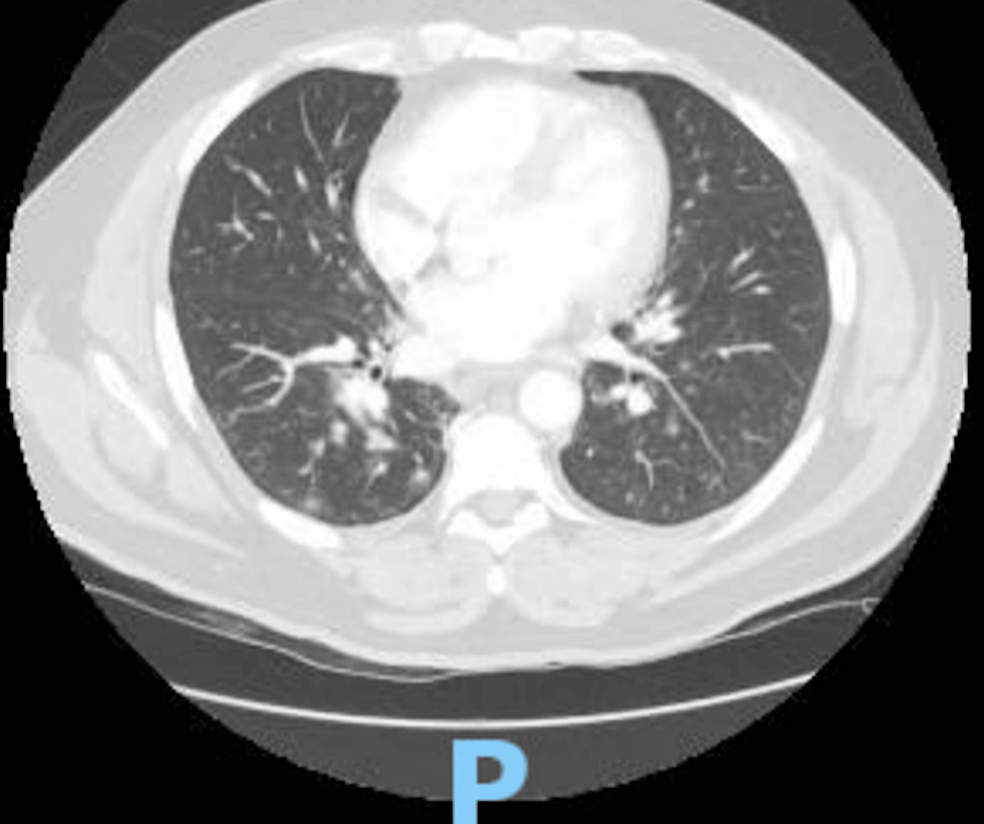
CTA scan of the chest performed on hospital day two to rule out acute pulmonary embolism demonstrating patent central airways, numerous patchy bilateral lower lobe predominant nodules/opacities.

**Figure 3:**
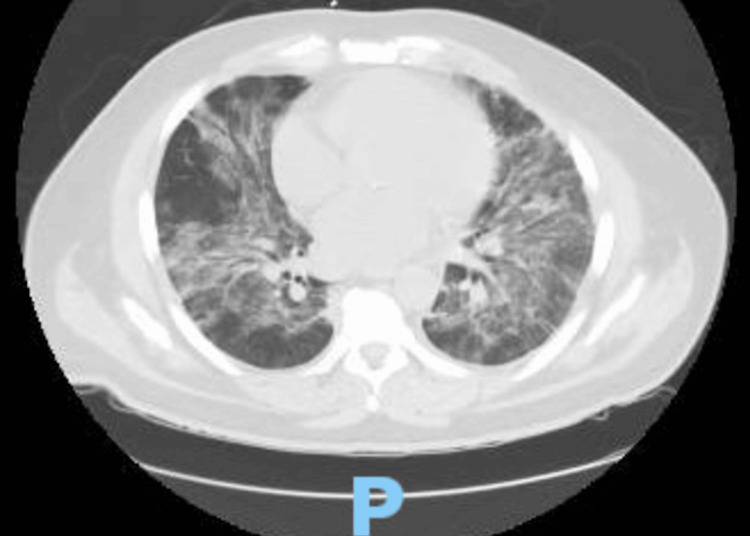
Repeat CT chest performed on hospital day seven to evaluate the patient’s respiratory status, which demonstrated increased bilateral ground glass and consolidative opacities, concerning worsening pneumonia.

A 60-year-old male, with a past medical history of hypertension, hyperlipidemia, and exposure working as a firefighter in the aftermath of the terror attack in New York City on September 11, 2001 (for which he received annual cancer screening), presented to the hospital for a one-month history of generalized malaise. Before admission, he was diagnosed with sinusitis and started a course of azithromycin. During his treatment, he developed a papular, non-painful, non-pruritic rash along with flat, flesh-colored patches on his trunk, sparing his face, neck, and extremities, followed by a five-day history of fevers and chills, measuring a maximum of 103°F at home and shortness of breath, prompting his presentation to the emergency department. On admission, the patient presented with normal vitals: BP of 124/64 mmHg, heart rate of 81 bpm, respiratory rate of 17 breaths per minute, and oxygen saturation of 95% breathing ambient air. The initial workup included an ECG, demonstrating sinus rhythm with a low-voltage QRS and shortened PR interval (Figure 1.) Given the dyspnea, there was an initial concern for a pulmonary embolism. While the initial computed tomography (CT) angiogram of the chest (Figure 2) demonstrated no pulmonary embolism, it did reveal numerous patchy bilateral lower lobe predominant opacities/nodules and multiple prominent indeterminate upper abdominal lymph nodes. The patient's complete blood count throughout admission is listed in the table below (Table 1). His complete metabolic panel was notable for transaminitis, demonstrating an ALP of 143 IU/L, AST of 44 IU/L, and ALT of 55 IU/L. Procalcitonin was notable at 1.17 ng/mL, and C-reactive protein was elevated at 425.5 mg/L. Initial urinalysis and urine culture were negative. Troponins drawn at the time of admission were <0.1 ng/mL. Given the patient's fever, relatively low oxygen saturation, and CT findings, the patient was diagnosed with pneumonia and admitted to the general medicine service, where he was started on empiric antibiotic treatment with vancomycin, cefepime, and azithromycin. Despite continued treatment, including completion of an empiric course of antibiotics, his respiratory and mental status both acutely worsened. Diagnostic evaluation for infectious etiology was negative, including blood cultures, respiratory viral panel, and urinary antigens for Legionella and Pneumococcus. By hospital day five, the patient required an ICU upgrade for worsening dyspnea on non-invasive mechanical ventilation. His lymphocytosis was noted in the initial blood work; however, as the WBC count was down-trending, it was attributed to infection versus an adverse reaction to the outpatient antibiotics that the patient had been taking for sinusitis. However, the persistence of abnormal blood counts raised suspicion for a hematological disorder. Hematology/oncology was consulted for leukocytosis and thrombocytopenia. A peripheral blood flow cytometry demonstrated blasts of 12% and an increase in monocytes (57%) with immature forms. A repeat chest CT performed on hospital day seven for worsening respiratory status (Figure 3) demonstrated increased bilateral ground glass and consolidative opacities concerning worsening pneumonia. On hospital day nine, a bone marrow biopsy was performed, revealing hypercellular (>95%) marrow, and was replaced mainly by blasts/blast equivalents with minimal normal trilineage hematopoiesis. Concurrent flow cytometry (76-FL-23-5902) revealed an aberrant myeloblast population (19%) and monoblastic/monocytic population (21%). PML-RARA FISH (76- FH-23-98572) is negative. These findings confirmed the diagnosis of AML with monocytic differentiation. The patient was eventually transferred to another hospital to initiate therapy for AML.

**Table 1:** CBCs throughout admission.

Date	Hospital Day 1	Hospital day 2	Hospital day 3	Hospital day 4	Hospital day 5	Hospital day 6	Hospital day 7	Hospital day 8
WBC count	21.52 cells /µL	84.05 cells /µL	49.98 cells /µL	37.43 cells /µL	17.66 cells /µL	16.59 cells /µL	19.01 cells /µL	9.98 cells /µL
RBC count	4.89 million/mm^3^	4.06 million/mm^3^	3.85 million/mm^3^	3.92 million/mm^3^	4.22 million/mm^3^	3.59 million/mm^3^	3.74 million/mm^3^	3.47 million/mm^3^
Hemoglobin	15.2 g/dL	12.8 g/dL	11.8 g/dL	12.4 g/dL	13.3 g/dL	11.6 g/dL	11.7 g/dL	10.7 g/dL
Hematocrit	43.5	36.6	34.2	35.0	37.5	31.6	32.7	29.7
MCV	89.0 µm^3^	90.1 µm^3^	88.8 µm^3^	89.3 µm^3^	88.9 µm^3^	88 µm^3^	87.4 µm^3^	85.6 µm^3^
Platelet count	92 cell/mcL	92 cell/mcL	61 cell/mcL	55 cell/mcL	50 cell/mcL	44 cell/mcL	58 cell/mcL	61 cell/mcL
Neutrophil %	21.5		8.5	27.0	17.7	20.0	20.0	56.2
Lymphocyte %	19.6		18.6	22.0	17.9	14.9	2.84	17.9
Monocyte %	53.1		70.5	44.0	62.0	59.1	59.1	18.7
Eosinophil %	2.0		0.2	0.1	0.1	0.1	0.1	0
Basophil %	0.6		0.3	0.2	0.2	0.04	0.4	0

## Discussion

The clinical trajectory of this patient highlights the diagnostic challenges when AML presents with acute respiratory failure suggestive of pneumonia. The overlap of symptoms between AML and respiratory distress mandated a nuanced diagnostic approach. Considering that the patient's medical history did not include any hematologic disease, his ongoing condition was initially attributed to an infectious process, and all interventions were focused on managing an infectious disease and its complications. 

Initially, the patient's presentation with fever, leukocytosis, thrombocytopenia, transaminitis, and hemodynamic instability naturally steered the diagnostic focus toward infection. However, as the patient's condition failed to improve despite aggressive antimicrobial treatments and a proper history elicited his involvement in 9/11 rescue operations, a shift in diagnostic direction became imperative. The persistence of constitutional symptoms despite antimicrobial interventions added suspicion to our thought that this could be a malignancy, prompting a reevaluation of the running diagnoses. The comprehensive infectious workup, while necessary to eliminate microbial sources, also played a role in redirecting the investigative pathway.

Pulmonary infiltration by leukemia cells is commonly observed in the advanced stages of both acute and chronic leukemia [4]. Nevertheless, the occurrence of symptomatic pulmonary infiltrates as the primary presentation of acute leukemia is infrequent [5]. Acute monocytic leukemia is associated with a high risk of leukemic pulmonary infiltration. Azoulay et al. reported 20 patients with acute respiratory failure related to leukemic pulmonary involvement from leukostasis or leukemic infiltration. All 20 patients had acute respiratory failure as the presenting manifestation of acute leukemia. All patients had the same type of acute myeloid leukemia involving monocytic cells [5].

## Conclusions

The early involvement of hematologic-oncologic expertise, prompted by evolving leukocytosis and monocytosis, allowed for a bone marrow biopsy and flow cytometry to be obtained prior to discharge - ultimately establishing the diagnosis. The trajectory of AML presenting as an infectious syndrome underlines the need for meticulous diagnostic exploration, especially when clinical manifestations belie the deeper pathology. Interdisciplinary collaboration, combining clinical acumen with various diagnostic tools, emerges as the linchpin in deciphering such enigmatic clinical scenarios.
